# Automatic cell counting from stimulated Raman imaging using deep learning

**DOI:** 10.1371/journal.pone.0254586

**Published:** 2021-07-21

**Authors:** Qianqian Zhang, Kyung Keun Yun, Hao Wang, Sang Won Yoon, Fake Lu, Daehan Won

**Affiliations:** 1 Department of System Science and Industrial Engineering, State University of New York at Binghamton, Binghamton, NY, United States of America; 2 Department of Biomedical Engineering, State University of New York at Binghamton, Binghamton, NY, United States of America; Taipei Medical University, TAIWAN

## Abstract

In this paper, we propose an automatic cell counting framework for stimulated Raman scattering (SRS) images, which can assist tumor tissue characteristic analysis, cancer diagnosis, and surgery planning processes. SRS microscopy has promoted tumor diagnosis and surgery by mapping lipids and proteins from fresh specimens and conducting a fast disclose of fundamental diagnostic hallmarks of tumors with a high resolution. However, cell counting from label-free SRS images has been challenging due to the limited contrast of cells and tissue, along with the heterogeneity of tissue morphology and biochemical compositions. To this end, a deep learning-based cell counting scheme is proposed by modifying and applying U-Net, an effective medical image semantic segmentation model that uses a small number of training samples. The distance transform and watershed segmentation algorithms are also implemented to yield the cell instance segmentation and cell counting results. By performing cell counting on SRS images of real human brain tumor specimens, promising cell counting results are obtained with > 98% of area under the curve (AUC) and *R* = 0.97 in terms of cell counting correlation between SRS and histological images with hematoxylin and eosin (H&E) staining. The proposed cell counting scheme illustrates the possibility and potential of performing cell counting automatically in near real time and encourages the study of applying deep learning techniques in biomedical and pathological image analyses.

## Introduction

Identification and counting the number of cells is one of the major tasks for biomedical image analyses and medical diagnoses [1]. Cell density estimation, which can be obtained by counting the number of cells within a certain region of the image, is an essential hallmark feature with a high correlation to medical diagnostic results [2, 3]. An accurate estimation of cell density can promote the diagnosis and grading of tumors, enable a precise definition of tumor biopsy target, facilitate therapeutic decision making, and assist surgical planning [4]. In particular, cell counting is conducted for brain tumors in this research because it is one of the most dangerous and deadliest cancers due to the aggressive and heterogeneous nature, which leads to a relatively low survival rate. The survival rate of malignant brain tumor is 35% [5]. The primary brain cancer cells are the cancer cells that can conduct uncontrollable cell division within or around the brain, which impacts brain functions and results in disability. In addition, the group of abnormal cells can affect the health of other brain cells [6]. Death usually occurs if a brain tumor is severe or on a critical position within the brain. There are more than 100 types of brain tumors [7], e.g., Meningioma, Pituitary, Glioma, etc. [8]. Based on the malignancy, cell distribution characteristics, and spread speed, brain tumors can be graded into four malignancy grades by the World Health Organization (WHO) [7, 9]. A higher grade indicates a higher malignancy level. If a brain tumor is diagnosed as grade 3 or 4, it is considered a malignant one.

It is important to provide precise therapy treatment for brain tumors. The main treatment for malignant brain tumors is surgical gross total resection [10], which requires a precise analysis of tumor regions and margins due to the complex structure of the brain. Among the variant medical imaging techniques, magnetic resonance imaging (MRI) is used as the standard process during brain surgeries because there is no radiation involved and clear imaging of soft tissues can be provided. However, MRI images suffer from the inaccuracy of tumor boundary detection [11]. Hematoxylin and eosin (H&E)-stained cryosection is often implemented for intra-operative diagnosis [12]. The limitation of H&E imaging is that obtaining a microscopic review of frozen tissue is time-consuming (30 minutes) and labor-intensive with a high cost, which limits its wide application to provide brain tumor diagnostic guidance before or during the tumor resection process [13].

Recently, label-free neuropathological imaging of brain tumor tissue that uses the stimulated Raman scattering (SRS) microscopy has been demonstrated [3]. SRS neurosurgical pathology images can be generated in real time by a rapid mapping process of lipids and proteins from a fresh specimen. SRS is a third-order nonlinear optical process. In SRS imaging, two ultrafast laser beams are used to excite the sample. The frequency difference between the two laser beams is tuned to a particular Raman shift for imaging. SRS signal is linear to the Raman signal with much higher intensity by a few orders of magnitude and therefore enables rapid Raman imaging. SRS images allow pathologists to capture the fundamental diagnostic hallmarks from fresh brain tumor tissue that cannot be extracted by other medical imaging modalities. The advantages of SRS for brain tumor diagnosis include i) rapid identification of white and gray matter, ii) cell body visualization, iii) vascular proliferation identification, iv) necrosis and viable tumor discrimination, and v) visualization of both tumor cells and the extracellular matrix components [3].

Owing to the rapid mapping of lipids and proteins using SRS microscopy, cells that consist mainly of protein can be identified [14]. However, the cell contrasts of SRS are weaker than other histological image modalities, e.g., H&E-stained images. The weak contrast of cells from SRS images, along with the limitation of high time cost and subjective errors, makes it challenging to perform manual counting. The development of clinical data collection technologies and artificial intelligence (AI) leads to an improvement of computer-aided diagnosis (CAD) systems, which provides complementary analyses of clinical data and enhances the disease diagnosis process [15, 16]. Taking advantage of AI and machine learning (ML), much research has integrated various ML approaches into CAD systems [17–19]. The potential of applying AI and ML on medical image analysis has been illustrated by providing reliable diagnosis results and enhancing analysis effectiveness. In terms of SRS image analysis, high reliance on the ratio of lipid/protein ratio may cause the obtained images to suffer from noises generated by strong protein or lipid signals. For instance, blood vessels and regions of microhemorrhages during the biopsy procedure would develop noises in the images. Despite the substantial advantages of SRS images in regard to capturing the distinct characteristics of brain tumors, especially gliomas [3], the cell contrasts of SRS are weaker than other medical image modalities. Also, the heterogeneity of cellular morphology and the wide range of cell sizes essentially aggravate the challenges of cell counting. Moreover, the lack of cell annotation information leverage the challenge of performing the pixel-level model training.

In this research, the task of automatic cell counting and density estimation from SRS images for brain tumors is addressed through a deep learning-based cell counting framework. Specifically, an effective medical image semantic segmentation model, U-Net [20], is applied and modified to segment cells from the brain tumor samples by using a small number of annotations. To deal with the large image size of the brain tumor caused by the high resolution, i.e., 0.37 *μm*/*pixel* or 0.18 *μm*/*pixel*, we adopted a split-and-combine scheme, which performs cell segmentation based on cropped small patches and then combines patch segmentation results accordingly. Based on the cell segmentation results, the distance transform and watershed segmentation algorithms are implemented to achieve the cell instance segmentation, which therefore results in cell counting. To evaluate the cell counting results from the SRS images, cells are also counted using the similar approach from the paired H&E images, with the only exception that an unsupervised ML method, K-means clustering, is employed to segment cells so that the cells can be segmented without requiring segmentation labels. The cell segmentation and counting on real brain tumor SRS samples obtains promising results with > 98% of area under the curve (AUC) and *R* = 0.97 in terms of cell counting correlation between SRS and H&E. The main advantage of the proposed framework is that pixel-level real-time cell segmentation and automatic cell counting can be achieved using only limited training samples, given the existence of SRS image noises and the cell morphology heterogeneity. black This research not only demonstrates the possibility of performing SRS image analysis in a much more detailed level but also enhances the potential of promoting the SRS technique into the surgical process, which quickly provides surgical guidance without the requirement of the time-consuming staining process. The main contributions of this research are summarized as follows:

1. A cell counting framework is proposed for high-resolution brain tumor SRS images by adopting a split-and-combine scheme.
2. The U-Net model is modified and applied to segment cells efficiently from the brain tumor samples for SRS images by using a small amount of annotation information.
3. The K-means clustering is employed to segment cells for H&E images so that cells can be segmented without requiring segmentation labels. In particular, the cells segmented from H&E images can serve as the reference for the evaluation of cell counting from SRS images.
4. The distance transform and watershed segmentation algorithms are implemented to achieve the cell instance segmentation and therefore cell counting results.

The rest of this paper is organized as follows: Related cell counting research is reviewed in Section 2; Section 3 describes the proposed cell counting framework for SRS and H&E images; Section 4 discusses the experimental results and analysis; Conclusions and future work are shown in Section 5.

## Literature review

In recent years, the deep learning technique has brought promising performance to various image analysis tasks, such as classification, detection, segmentation, etc [21]. Most research in the literature focuses on the design of convolutional neural network (CNN) models for natural images, mainly due to the availability of large public datasets, e.g., ImageNet [22]. The fast-growing computational speed and capacity also facilitate the attempts of different CNN model structures and model training strategies. The idea of CNNs started in 1989 based on the structure of artificial neural networks (ANNs), backpropagation, and the introduction of convolutional layers [23, 24]. Then, the first CNN model, named LeNet5, was designed in 1998 [25]. The CNN study was suspended due to the limitation of hardware and memory capacity until another breakthrough in 2012: AlexNet [26]. AlexNet includes the rectified linear unit (ReLU) activation function, dropout concept, local response normalization (LRN), along with the application of data augmentation. Since then, the CNN technique has been extended to various structures with different applications. Some studies focus on increasing the depth of CNNs. VGG-16 and VGG-19 were proposed with 16 and 19 layers, respectively [27]. To solve the problem of vanishing and exploding gradients due to the increased depth of CNN models, a residual mapping process was introduced by ResNet [28], which improved the model effectiveness. The depth of CNN models can be more than 1,000 layers in 2016 [29]. Some other studies focus on reducing computation complexity. The development of GoogleNet [30] brought the idea of inception layers, which dramatically reduced the number of parameters while keeping the same receptive fields through the combination of various kernel sizes. Based on the concept of GoogleNet, some other deep learning models were designed, including Inception V2, Inception V3 [31], Inception V4 [32], and Xception [33]. Densely connected convolutional networks (DenseNet) were also proposed, which concatenate all layers using the residual mapping from all preceding layers [34]. The development of various deep learning models essentially boosts the research of different practical problems, such as image denoising [35], super-resolution [36], image registration [37], image reconstruction [38], human authentication [39], etc.

In addition, SRS has become an emerging technique for intraoperative histology analysis, which leads to the attempt to incorporate deep learning into SRS image analysis, especially tumor diagnosis [40]. Stimulated Raman histology (SRH) was employed to generate virtual H&E-like images. Then, multilayer perceptron (MLP) and random forest were applied to predict lesions from tissue patches [41, 42]. Alternatively, tumor classification can be directly performed based on SRS images. To achieve accurate diagnoses of laryngeal squamous cell carcinoma, a 34-layer ResNet model was applied to classify normal and neoplastic larynx tissues from SRS patches [43]. LeNet5 was applied to recognize prostate cancer patients with bone metastases using surface-enhanced Raman spectroscopy (SERS) images [44].

Regarding cell counting, it can be categorized as detection-based counting and regression-based counting [18]. Detection-based cell counting involves the detection or segmentation of every single cell prior to cell counting, which requires a supervised learning process. In such an approach, cell annotation information is needed to train the detection or segmentation model, which converts the counting task to a detection task. The annotation information could be dot annotation of objects [18, 45, 46], bounding boxes around the objects [47], etc. Each cell is detected and localized one by one through the object detection model, and a counter then takes the detected cells and yields the count results [48]. A typical cell detection work in Arteta et al. (2012) detected cells using a three-step approach: 1) cell-like candidate region identification that uses maximally stable extremal regions (MSER) detector; 2) candidate region evaluation that uses support vector machine (SVM); and 3) non-overlapping region selection that uses dynamic programming [49]. Other research follows a similar pipeline that counts objects based on detection results [50–52].

Currently, more efforts have been made to count cells by regression, which avoids the challenging task of detection or segmentation of single cells and generates cell density or cell count directly from input images [18, 48]. The CNN models such as deep residual networks were applied and modified using the Euclidean loss function by taking the total number of cells as the annotation information [48]. An ensemble of regression trees that uses dense features to estimate the object density map by averaging structured, patch-based predictions was implemented [53]. Inspired by the success of fully convolutional networks (FCN) for image semantic segmentation, the FCN algorithm has been borrowed for cell counting. Xie et al. (2018) modified the FCN model such that the cell spatial density map can be predicted from dot-annotated microscopy images. The number of cells within a certain region was obtained via the integration over the density map. In this work, cells can be also detected as a side benefit from FCN [18]. Objects can be also counted following the same framework that is similar to the work of Xie et al. (2018), where a density map was obtained by formulating a minimization of a regularized risk quadratic cost function as a cutting-plane optimization problem [54]. In another research, a FCN-based framework was proposed, which consists of a primary FCN and a set of auxiliary FCNs that provide extra learning features from intermediate layers for the primary FCN. In addition, shortcut connections were integrated into the primary FCN, which can enhance the granularity of the features and density map estimation [1, 55]. Morelli et al. (2021) follow the similar concept that employs the FCN with short connections between convolution blocks to segment cells [56]. A residual dilated U-Net was proposed, along with the application of an ensemble method to count and localize blastomere cells [57]. Alternatively, a robust nuclei instance segmentation architecture was proposed, which includes multiple U-Net structures; one model detected and segmented cells and the other model refined segmentation results [58]. Moreover, in the research conducted by Villa et al. (2018), cells were counted dynamically using multiple frames by proposing a spatiotemporal model that employs CNN and long short-term memory (LSTM). Therefore, the cell count variation can be monitored over time [59]. A summary of related research for cell counting research is provided in Table 1.

**Table 1:** A summary of the cell counting research.

Category	Authors	Method
Detection-based	Arteta et al. (2012)	SVM, dynamic programming, MSER detector
	Arteta et al. (2016)	Tree-structured discrete graphical model, SVM, dynamic programming
	Hosseini et al. (2020)	Mask R-CNN
Regression-based	Xue et al. (2016)	AlexNet, ResNet
	Xie et al. (2018)	Fully convolutional regression network
	Rad et al. (2018)	Ensemble residual dilated U-Net
	Villa et al. (2018)	CNN, LSTM
	Xue et al. (2019)	CNN, compressed sensing, sparse coding
	Xu et al. (2019)	U-Net
	He et al. (2019, 2021)	FCN, shortcut connections

Regarding the SRS image analysis, there are some attempts in the literature that integrated the ML technique, specifically, deep learning, into the analysis. Most research performed the lesion prediction task for image patches within a specimen [41, 43]. This research extends the simple image classification to pixel-level analysis by providing the cell segmentation and cell counting results that can reveal the intrinsic sample characteristics of brain tissues. Motivated by the superiority of U-Net over FCN on the medical image semantic segmentation task, this research employs and modifies U-Net to segment cells on SRS images. Also, the proposed cell counting scheme enables a mix of detection and regression-based counting because cells are segmented without the requirement of identifying each cell instance, but identified and counted through the involvement of morphological analysis.

## Methodology

**Fig 1:**
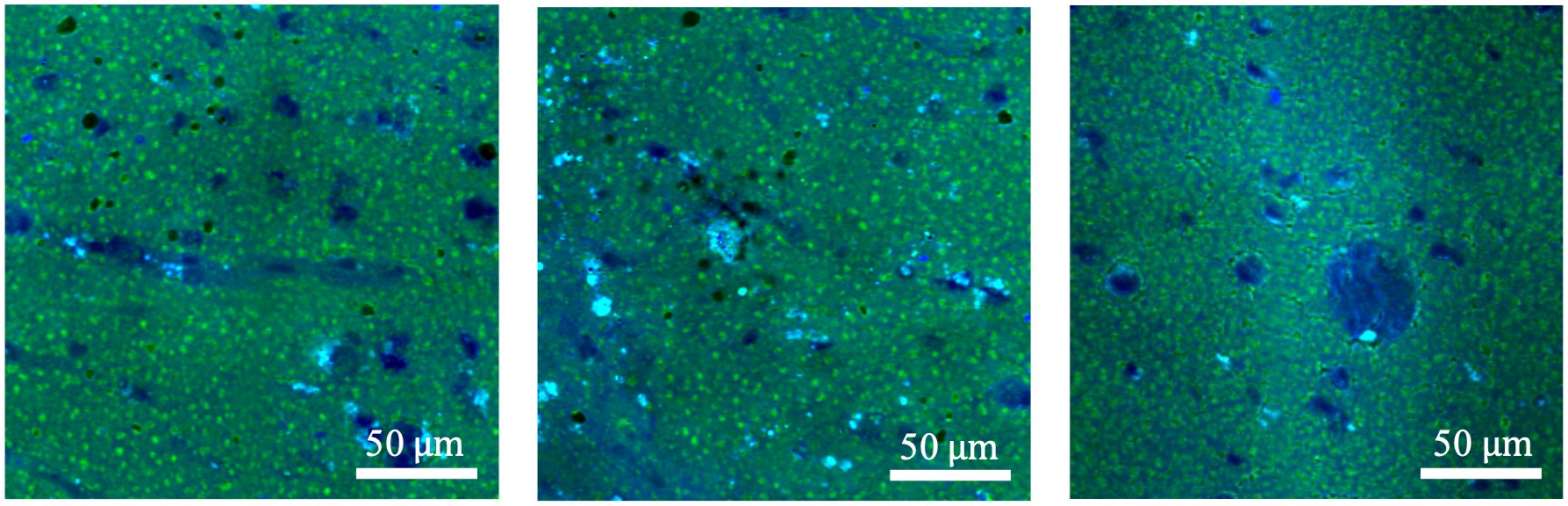
SRS samples. Representative SRS images show cell bodies embedded in the fatty tissue background. Pseudocolor green: lipids; Pseudocolor blue: proteins.

**Fig 2:**
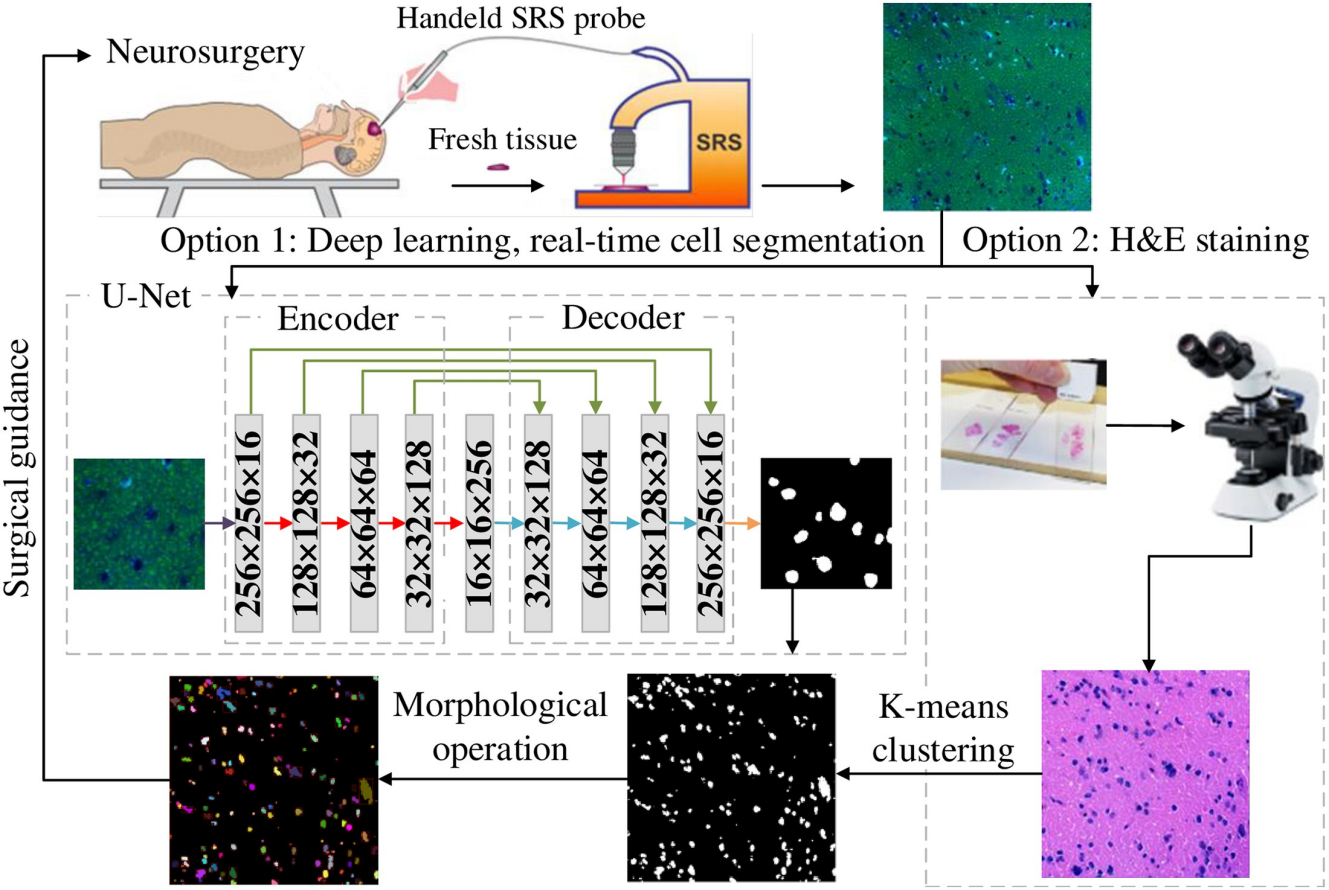
Overview of the cell counting framework.

In general, SRS images are collected via fresh biopsy samples taken from brain tissue and processed by Stimulated Raman imaging. Fig 1 shows SRS images from brain tumor samples. The cell bodies are shown with blue signals embedded in the fatty tissue background of green signals. Fig 2 shows an overview of the cell counting framework that can provide clinical support for image-guided brain tumor surgery in the operating room.

The proposed cell counting framework can be regarded as a hierarchical approach: 1) cell semantic segmentation and 2) morphological operation that recognizes distinct cell instances. In the first step of cell semantic segmentation, there are two machine learning-based options: deep learning near real-time segmentation with the application U-Net and K-means clustering, which takes more than 30 minutes to stain samples using H&E. Subsequently, using the distance transform and watershed segmentation algorithms, cell instances are identified and therefore counted, which can provide surgical guidance in the operating room.

### Cell segmentation using U-Net based on SRS images

**Fig 3:**
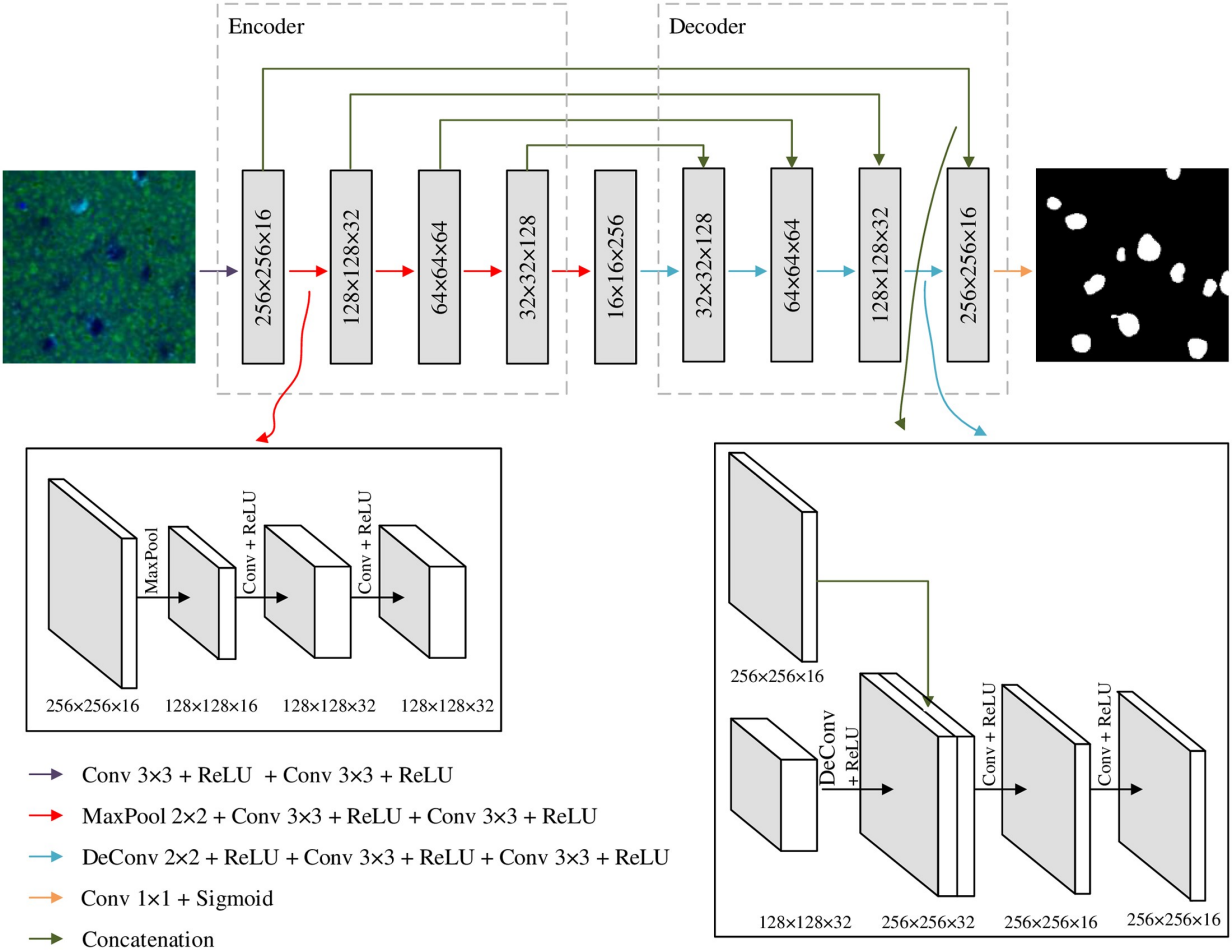
The architecture of the modified U-Net model.

The first approach processes the obtained SRS image from the biopsy samples through a split-and-combine method that performs cell segmentation based on cropped small patches and then combines patch segmentation results accordingly. Specifically, the SRS image is split into small patches with the size of 256 × 256 pixels, which essentially enlarges the number of samples for U-Net training and enables the model training from limited annotation information. In particular, U-Net is a state-of-art deep learning model proposed by extending the FCN model as a symmetric u-shaped architecture [20]. Instead of a single upsampling layer in FCN, U-Net utilizes multiple successive upsampling layers. To cope with the information loss problem due to the increase of successive layers, direct connections are built to propagate the context information to higher resolution layers. Consequently, U-Net consists of an encoder and a decoder, where the encoder applies the convolutional process and the decoder applies the deconvolutional process. The advantage of U-Net is that it can effectively segment objects from arbitrary size inputs. In addition, U-Net performs well with very few training images and achieves precise segmentation results, which leads to a wide application to solve medical image segmentation problems. Due to the relatively fixed color range of SRS images and limited training samples via manual annotation, we utilized half of the kernels compared to the original U-Net design. Therefore, there are 16, 32, 64, 128, and 256 kernels for the encoder and decoder in the five levels, which significantly reduce the model complexity and number of parameters to be optimized. Fig 3 shows the details of the U-Net implementation.

Different operations are represented via different colors. The input SRS samples are first convoluted through two successive 3 × 3 convolution operations with the ReLU activation function, which can improve the model computational efficiency and reduce the possibility of vanishing or exploding gradients. The next four layers follow a similar operation, with the exception that a 2 × 2 max pooling process is conducted to squeeze the information from the previous feature maps [60]. In the decoder, the feature maps are enlarged via a 2 × 2 deconvolution operation with the ReLU activation function, which are further concatenated with the feature maps from the encoder with the same sizes via direct connections. In such a way, the low-level feature information from the encoder can be propagated to the high-level feature maps in the decoder. Similarly, two successive 3 × 3 convolution operations with the ReLU activation function are attached to refine the cell characteristic extraction. Finally, the segmentation image is achieved through a 1 × 1 convolution process with one kernel by yielding a one-channel image. The Sigmoid activation function is employed, which yields the probability of each pixel to be predicted as a cell pixel. If the probability of a pixel as a cell is greater than 0.5, this pixel will be classified as a cell pixel. Otherwise, this pixel will be classified as a non-cell pixel. It is noted that because only blue and green channels are involved in the SRS images due to the nature of the SRS image generation process, the input images of the U-Net are two channels instead of the conventional RGB (red, green, and blue) color channels.

To optimize the U-Net model, the parameters are optimized by minimizing the cross-entropy between the prediction pixel labels and the annotated pixel labels. Backpropagation is used to calculate the gradient by defining 16 as the batch size. In each iteration, the parameters of feature maps are updated toward the optimal point via Adam, a first-order gradient-based optimization technique for stochastic objective functions with adaptive learning rate and gradient momentum estimation [61]. The default hyperparameters, step size *α* = 0.001, exponential decay rates for the first and second moment estimates *β*_1_ = 0.9 and *β*_2_ = 0.999, and a small number that prevents division by zero *ϵ* = 10^−8^, are used for the Adam algorithm. In addition, we implement the early stopping technique in the U-Net model training process to eliminate the case of overfitting. Specifically, 20% of the training samples are randomly selected as the validation dataset. During the segmentation model training, the loss function of the validation dataset is monitored whenever the whole training dataset has been utilized to update the parameters once, which is defined as one epoch. black If the validation loss has not been reduced for 25 epochs, the training process will stop. Then, the parameters with the least validation loss will be utilized as the final segmentation model.

### Cell segmentation using K-means clustering based on H&E images

The second method involves the H&E staining process, which requires at least 30 minutes, which thereby limits the practicability of applying such an approach to support the diagnosis and treatment processes in the operating room. However, the paired H&E brain sample images can serve as a reference to evaluate the cell counting results using the SRS images. Therefore, the H&E images are also analyzed via a clustering method, which aims to group the pixels from the H&E image into groups by means of recognizing statistically similar clusters. K-means clustering has been applied to the medical image segmentation problem as an unsupervised learning algorithm with the advantages of efficient calculation and ease of understanding [62]. Applying K-means clustering, the color values of pixels are used as the inputs. The objective is to minimize the sum of the squared similarity between all pixels to their corresponding cluster centroids [63, 64]. The objective function of clustering samples *X* = {*x*_1_, …, *x*_*n*_} with *K* clusters is

$$\begin{array}{c} J\left(C\right)=\sum_{k=1}^{K}\sum_{x_{i}\in c_{k}}^{}\left|\right|x_{i}-\mu_{k}{\left|\right|}^{2} \end{array}$$ (1)

where *x*_*i*_ − *μ*_*k*_ is the similarity between *x*_*i*_ and *μ*_*k*_, and *μ*_*k*_ is the centroid of cluster *k*. Here, the Euclidean distance is implemented as the similarity measure.

### Cell counting via morphological analysis

**Fig 4:**
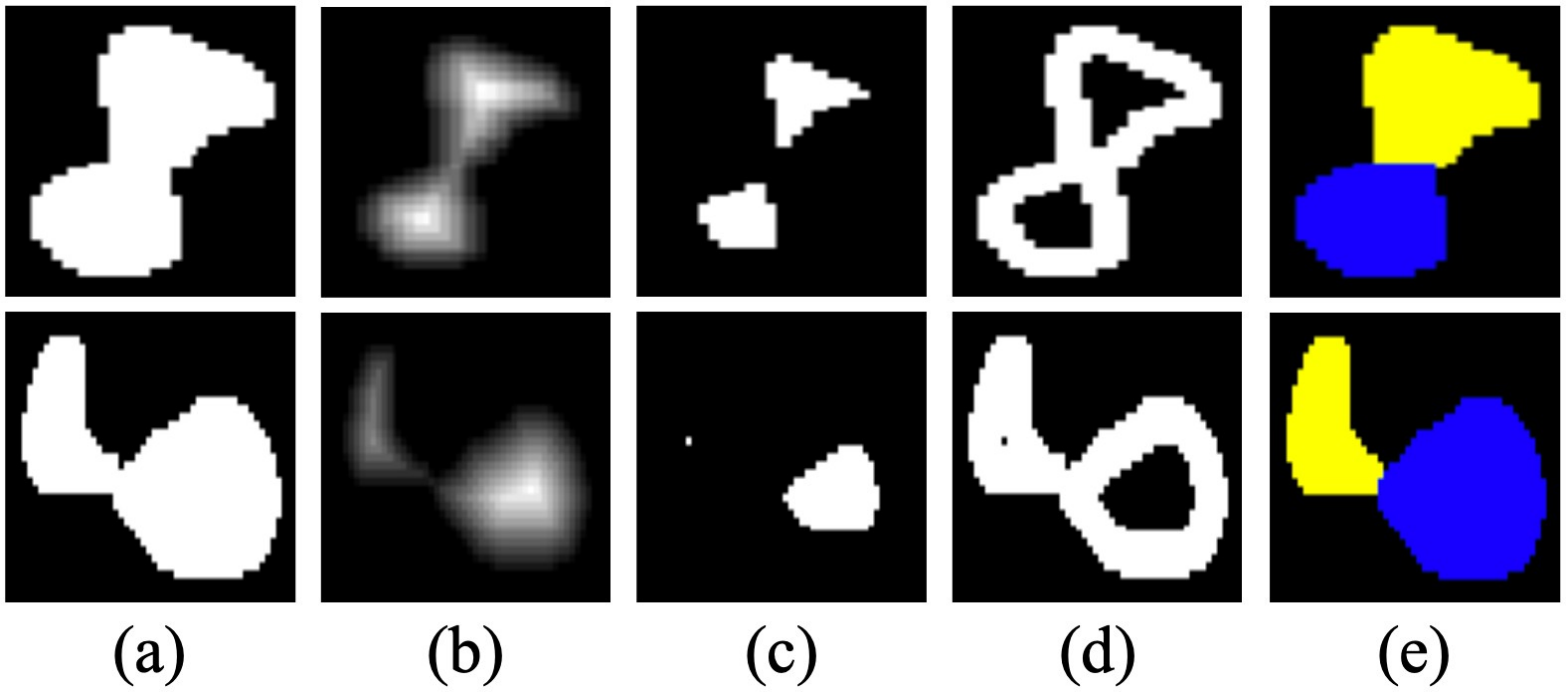
A demonstration of the distance-based watershed cell segmentation. A: Initial cell segmentation. B: Distance transform. C: Identified cell instance regions; D: Unknown region to be assigned. E: Final cell segmentation results.

Based on the cell segmentation results from both SRS and H&E images, cells are counted by identifying distinct regions. Given a cell segmentation image, a morphological opening operation is first performed to eliminate small dots, which are usually noises from the segmentation results. Subsequently, all the connected regions are identified and labeled as the initial cell instances by applying the OpenCV toolbox. black However, overlapping cells exist. In this case, multiple cells can be recognized as one region. Therefore, a post-morphological analysis that uses distance transform and watershed segmentation algorithms is further employed for each identified region, where connected cells can be split, which enhances the cell counting results [65]. Fig 4 presents two samples of connected cells, which are further split using the distance-based watershed segmentation method.

For each identified cell region, a distance map is generated via distance transform, which computes the minimum Euclidean distance from every pixel of the cell region to a background pixel, as shown in Fig 4(b). Following a rescale process that converts all non-zero distances between 0 and 1, a filter with the threshold defined as 0.5 yields the identified cell instance regions. The remaining unknown region is assigned to the identified cell regions using the watershed segmentation algorithm. Watershed was initially defined as the ridgeline that divides different areas drained by different river systems. Inspired by watersheds in geography, the watershed segmentation algorithm in mathematical morphology is designed by considering the image as a topographic landscape with ridges and valleys [65]. The objective of watershed segmentation is to trace all pixels toward a local minimum along the steepest descent direction. In this research, the unknown region pixels are assigned by using the negative of the obtained distance map as the evaluation criteria. As shown in Fig 4(e), the pixels are grouped according to the paths to their local minimum, which is also known as a catchment basin. In this way, each catchment basin refers to a cell instance.

A noise reduction process that defines thresholds of connected regions further eliminates noises. In particular, the regions with a size of less than 0.37 *μm* are excluded. Also, strong protein or lipid signals, such as blood vessels and microhemorrhages, can generate noises by showing cell-like color representations in SRS images. Usually, those blood vessels and microhemorrhages have large body sizes, which can be removed via a filter operation. If a region in a SRS image has the size of more than black100 *μm* or the area is more than black500 *μm*^2^, this region is excluded in both SRS and H&E images. To cope with the overlapped cells that cannot be split using the watershed segmentation algorithm, the identified cells with the body size larger than the upper inner fence of the cell sizes are counted as multiple cells by dividing the average cell size. black In this case, the maximum cell obtained is 375 *μm*^2^.

## Experimental results

In the experiment, brain tumor image samples are obtained from Lu et al. (2016) [3]. In particular, the brain tissues were collected from the Brigham and Women’s Hospital and Dana-Farber Cancer Institute. A flash-freezing process was conducted at -80°C, followed by a sectioning process to 12-*μm* thicknesses. The brain tumor samples are imaged by SRS and then stained using the H&E technique. A non-neoplastic benign brain tumor specimen with epilepsy and a malignant anaplastic oligodendroglioma specimen are utilized to conduct the cell counting task. Specifically, the resolution for the two specimens are 0.37 *μm*/*pixel* and 0.18 *μm*/*pixel*, respectively.

**Fig 5:**
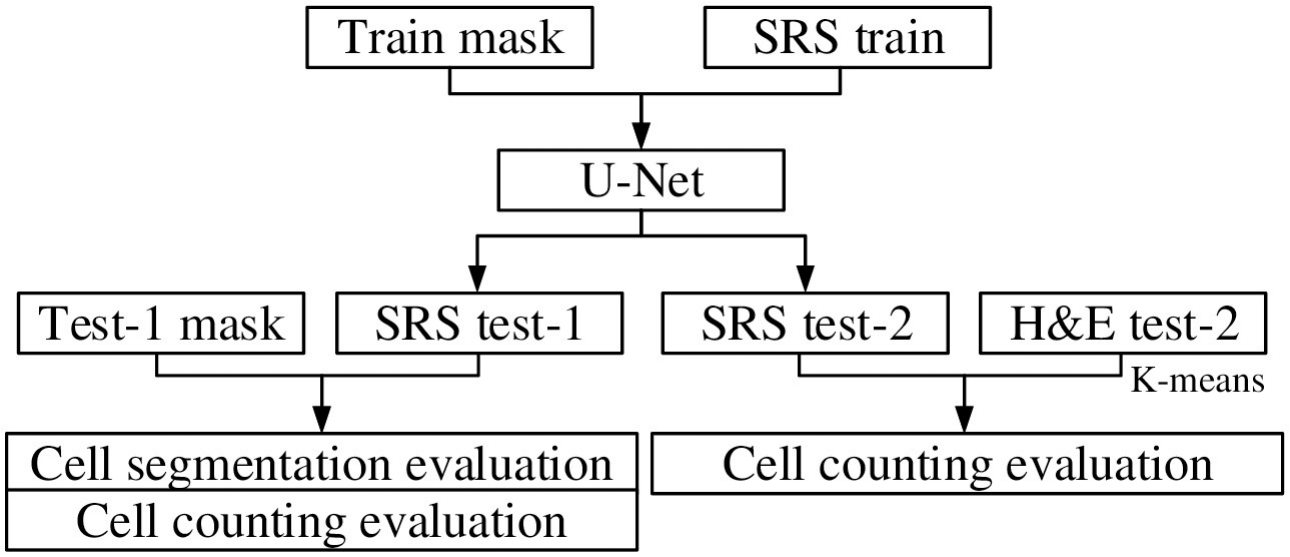
Cell counting framework. Cell counting framework. Train mask: manually generated cell annotation mask image for the training region; SRS train: the SRS image of the training region; Test-1 mask: manually generated cell annotation mask image for the first testing region; SRS test-1 and SRS test-2: the SRS images of the first and second testing regions; H&E test-2: the H&E image of the second testing region.

Fig 5 shows the cell counting framework in the experiments. The SRS image is split into three regions: one training region and two testing regions. It is noted that there are mismatches between the obtained H&E and SRS images for the same specimen regarding the cell shape, size, and position. Cell shiftiness and vanishing are also observed during the image collection process, which leads to a lack of ground truth cell distribution information. Therefore, the cells within the training region and the first testing region are annotated manually, which can be used to train the U-Net model and evaluate the cell segmentation results. To facilitate the U-Net training and testing, the training and testing regions are split into 256 × 256-pixel patch samples. The numbers of patch samples obtained from each region for the two specimens are presented in Table 2.

**Table 2:** Number of patch samples in each region.

	Specimen 1	Specimen 2
Train region	270	240
Test-1 region	120	100
Test-2 region	3536	1122

The cells in the two testing regions are segmented via the trained U-Net model. Then, the segmentation results are combined according to their original positions in the specimen. Having the manual segmentation mask in the first testing region, the cell segmentation and cell counting results are evaluated in the first test. Due to the lack of cell annotation in the second testing region, the corresponding region in the H&E image is segmented through K-means clustering. To promote the H&E image segmentation efficiency, the H&E image is split into 500 × 500-pixel patch samples, prior to the clustering for each patch sample. The cluster that has the minimum distance to cells in terms of pixel color, which is represented by dark blue in the H&E image, is identified as cells. By defining six clusters, the cells in the H&E can be well segmented. Also, the cell counting results from SRS and H&E images are obtained using morphological analysis and further compared.

### Pixel-wise evaluation on the first testing region

Utilizing the manual segmentation mask in the first testing region, the model evaluation adopts pixel-wise comparison by segmentation accuracy, specificity, sensitivity, and AUC. In addition, another performance metric, Dice coefficient (DICE) [66], a spatial overlap index is applied in the model evaluation, which is defined as follows:

$$\begin{array}{c} \text{DICE}=\frac{2\text{TP}}{2\text{TP}+\text{FP}+\text{FN}} \end{array}$$ (2)

where TP, TN, FP, and FN are the number of true positive, true negative, false positive and false negative, respectively. Moreover, the performance of cell counting is evaluated through percentage error (PE) as follows:

$$\begin{array}{c} PE=\frac{|N_{p}-N_{t}|}{N_{t}} \end{array}$$ (3)

where *N*_*p*_ is the predicted number of cells and *N*_*t*_ is the true number of cells in the same image. Tables 3 and 4 summarize the mean and standard deviation (SD) of the pixel-wise segmentation evaluation for the two specimens. Here, the modified U-Net, which is implemented in the framework, is referred as M-UNet. The traditional U-Net without early stopping is U-Net The ROC curves on the first testing region for the two specimens using M-UNet are also shown in S1 Fig. To confirm the necessity of the U-Net structure, the simplified U-Net that removes one encoder block and one decoder block is performed as 7layer-UNet. Two blocks of both encoders and decoders are also excluded as 5layer-UNet in the experiment. Also, FCN, which is widely used in the literature, is performed with the same model architecture without the concatenation process.

**Table 3:** Cell segmentation results on the first testing region for specimen 1.

Performance	M-UNet		U-Net		7layer-UNet		5layer-UNet		FCN	
Metrics	Mean	SD	Mean	SD	Mean	SD	Mean	SD	Mean	SD
Accuracy(%)	97.69	0.23	97.62	0.11	97.82	0.10	97.84	0.05	97.83	0.11
Sensitivity(%)	85.57	1.79	81.43	1.73	82.41	3.24	82.50	1.95	83.40	2.14
Specificity(%)	98.48	0.33	98.69	0.13	98.84	0.25	98.86	0.13	98.79	0.23
AUC(%)	98.96	0.16	98.59	0.22	98.94	0.11	98.92	0.10	99.02	0.14
DICE(%)	81.99	1.36	80.96	0.92	82.43	0.81	82.61	0.55	82.74	0.54
Epochs	60.40	5.18	300.00	0.00	60.20	4.76	77.80	16.12	65.20	6.61
PE(%)	2.49	1.36	2.51	2.08	3.45	4.73	3.26	1.62	4.07	2.64

**Table 4:** Cell segmentation results on the first testing region for specimen 2.

Performance	M-UNet		U-Net		7layer-UNet		5layer-UNet		FCN	
Metrics	Mean	SD	Mean	SD	Mean	SD	Mean	SD	Mean	SD
Accuracy(%)	99.11	0.10	99.15	0.05	98.88	0.05	98.55	0.04	99.12	0.05
Sensitivity(%)	87.64	1.34	87.78	1.22	86.12	2.39	74.14	2.65	86.47	2.83
Specificity(%)	99.54	0.13	99.57	0.05	99.36	0.14	99.45	0.11	99.58	0.09
AUC(%)	99.62	0.09	99.66	0.09	99.58	0.07	99.21	0.08	99.55	0.13
DICE(%)	87.56	1.21	88.11	0.75	84.66	0.33	78.47	0.65	87.47	0.84
Epochs	78.40	13.24	300.00	0.00	95.00	24.05	144.40	12.76	115.40	14.36
PE(%)	3.35	2.41	4.16	2.52	8.90	2.68	4.86	4.15	4.39	3.63

At the pixel-level cell segmentation, it is observed that reliable segmentation results can be obtained via different models, especially high segmentation accuracy and AUC. In contrast, the traditional image processing software, such as ImageJ [67], is not able to extract the cell instances from SRS images. The segmentation accuracy, specificity, AUC, DICE from different models are similar. However, the extreme imbalance between the cell and non-cell pixels reveals that sensitivity, which depicts the capacity of identifying the cell pixels, is more important than other performance metrics. For Specimen1, the M-UNet achieves higher sensitivity than other models. The M-UNet model also outperforms other methods regarding PE, which means more than 97.5% of cells can be identified and counted successfully. For specimen 2, M-UNet and U-Net outperform other models regarding the sensitivity, which illustrates the necessity of the U-Net structure. Also, the M-UNet achieves lower PE than the conventional U-Net. Overall, the comparison of different cell segmentation models shows that the implemented M-UNet is the optimal option in the cell counting pipeline.

### Cell counting evaluation on the second testing region

**Fig 6:**
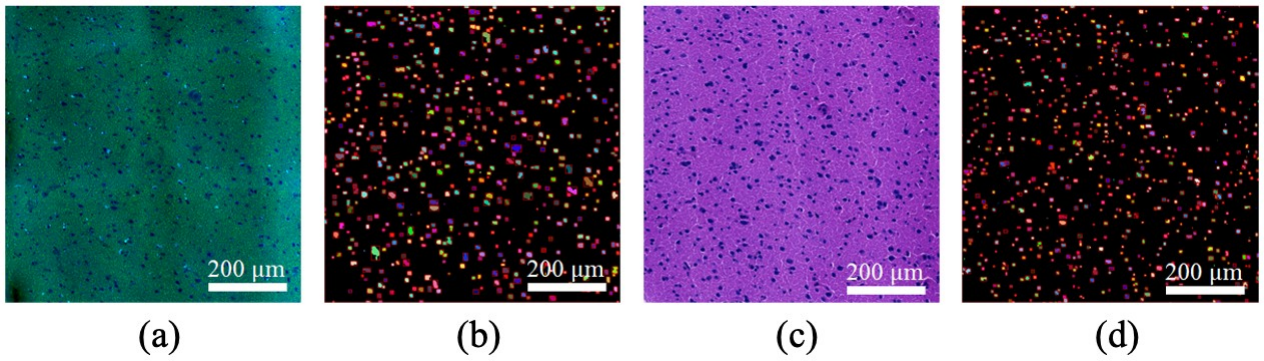
Cell segmentation and identification results in a FOV. A: SRS FOV. B: SRS Cell identification. C: H&E FOV; D: H&E Cell identification.

In the test on the second testing region, the cell counting results are further evaluated by comparing them to the H&E images. As cell shiftiness and vanishing are observed between the paired SRS and H&E images, it is not practical to perform the pixel-wise evaluation. To reduce the impact of cell shiftiness across patches, the testing images are split into multiple grids, named as fields of view (FOV), where each FOV consists of 8 × 8 patch samples with the size of 256 × 256 pixels for each patch. Therefore, the size of each FOV is 2048 × 2048. A sample of FOV from SRS and H&E images at the same position is shown in Fig 6.

**Fig 7:**
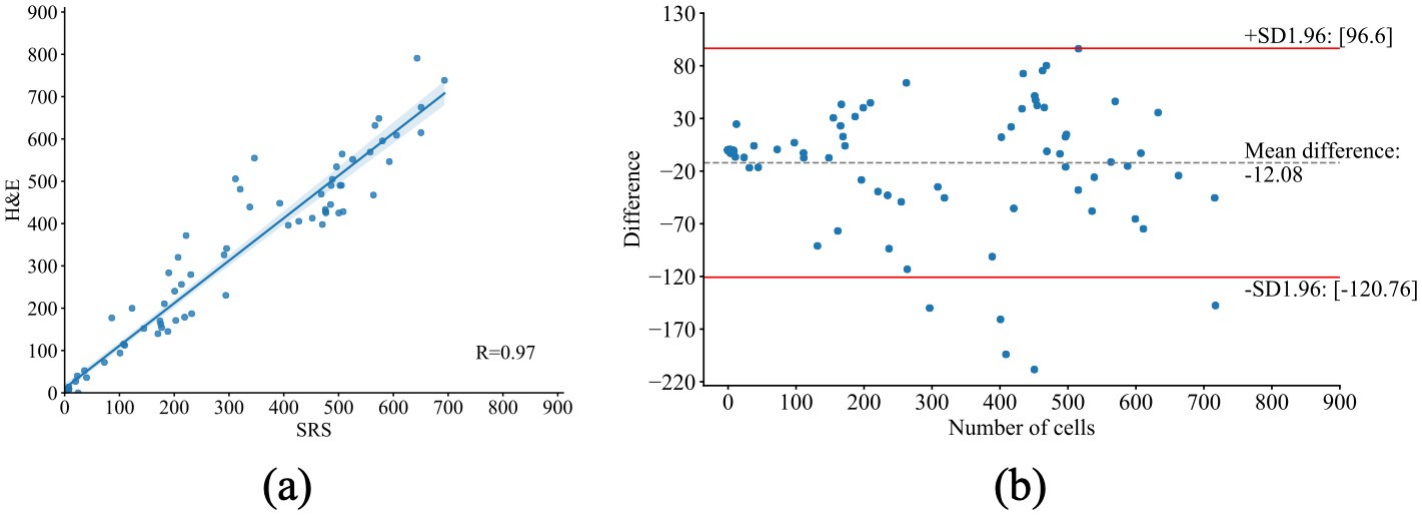
Plot of cell count from SRS and H&E images per FOV. A: Correlation plot. B: Bland-Altman plot.

The cell instances are identified and represented by different colors, which are obtained by counting the number of connected regions from semantic segmentation and distance-based watershed segmentation. Assuming the number of cells estimated from H&E images is the true cell density, the PE for the two specimens are 6.60% and 15.48%, respectively. Due to the internal difference between SRS and H&E images, such as cell shiftiness, the number of cells in the SRS images can be different but has a high correlation to the H&E images. The number of cells per FOV from both SRS and H&E images are plotted in Fig 7(a), which represents a high correlation (*R* = 0.97) between the two image modalities. Fig 7(b) is the Bland-Altman plot of the cell counting results, which shows the difference of the detected cells between SRS and H&E images for each FOV. It is observed that on average 12.08 cells in H&E FOVs are not detected in the paired SRS FOVs. Considering 95% confidence interval (CI) of limits of agreement (LoA), the cell counting results of 83 FOVs are within the LoA out of 88 FOVs. The five FOVs that observed fewer cells from SRS images all come from the first specimen in which there exists cell vanishing in SRS.

**Fig 8:**
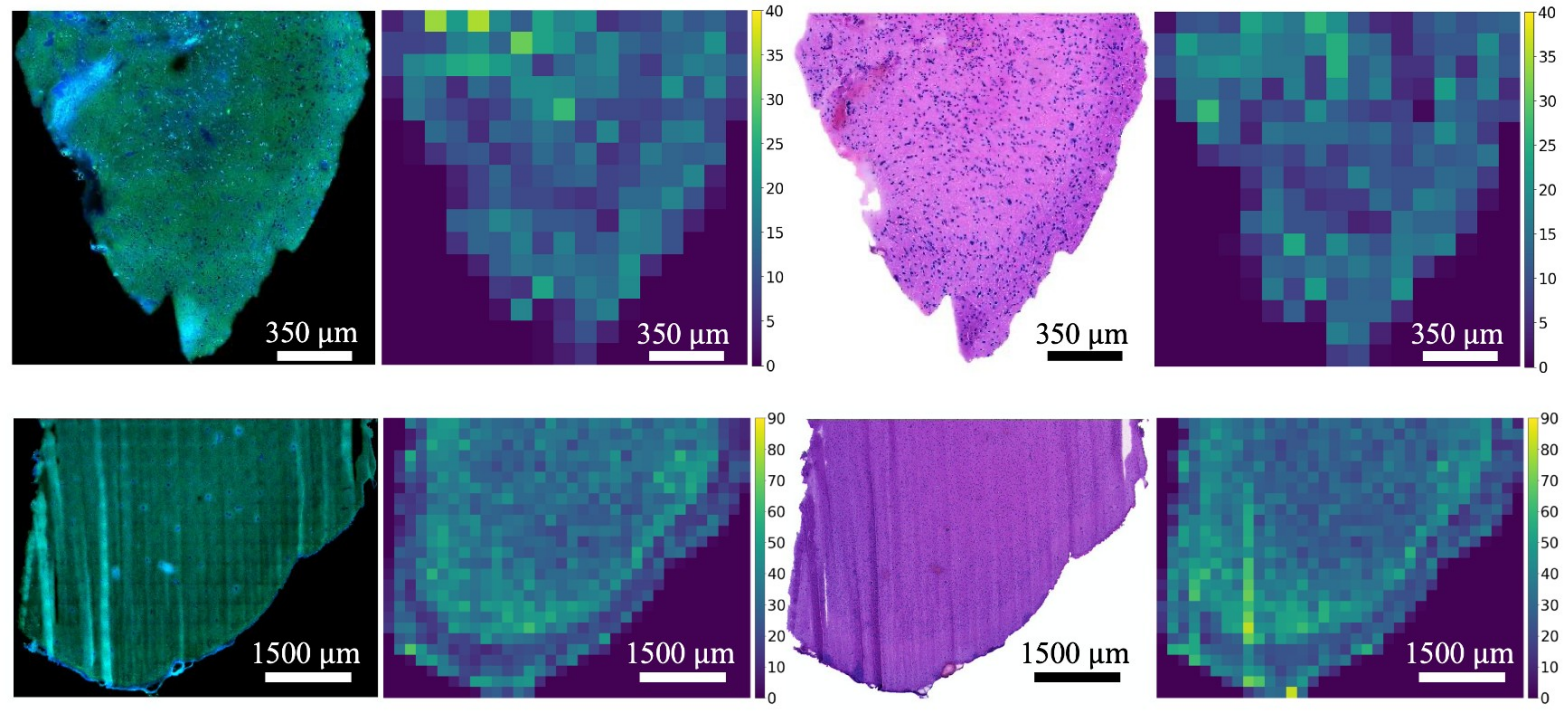
Cell density heatmaps of the second testing region for the two specimens. The first and third columns are SRS and H&E images; The second and fourth columns are the corresponding cell density heatmaps for SRS and H&E images.

To further visualize the cell density, the second testing regions are split into 512 × 512-pixel patches and the corresponding heatmaps are generated, as shown in Fig 8. Here, the number of cells for each patch is mapped, which thereby provides support for physicians and pathologists to easily understand the cell distribution within a specimen, conduct cancer diagnosis, and suggest surgery planning. It is observed that the heatmaps from SRS images maintain a similar distribution from H&E images, except for several patches caused by the internal cell distribution changes.

## Conclusion

This study aims to promote the implementation of AI to biomedical analysis for SRS images. Estimating tumor cell density is one of the key pathological hallmarks in the process of cancer diagnosis, and our work shows that this can be addressed by means of proposing an automatic deep learning-based cell counting scheme. To the best of our knowledge, this is the first time that deep learning-based cell counting is performed on SRS images. Using a two-step hierarchical process, cells are first segmented using the U-Net model that requires a small amount of training data. Based on the cell segmentation results, the distance transform and watershed segmentation algorithms are implemented to generate the cell instance identification and therefore cell counting results. According to the manual annotation on two brain tumor specimens, promising cell segmentation results are obtained with > 98% AUC. Also, comparing the cell counting results from the proposed deep learning model on SRS images to K-means clustering on H&E images, a linear correlation *R* = 0.97 is achieved. By providing cell density maps through reliable cell counting results, the possibility and practicality of conducting automatic cell counting are illustrated.

The main limitation of this research is that the employment of U-Net requires manually generated cell annotation, which is prone to subjective errors from weak cell contrast. The future research direction can be focused on unsupervised models, such as adversarial learning. The overlapped cells that cannot be split using the watershed segmentation algorithm also preclude accurate cell counting, which can be addressed by the use of more morphological techniques in the future. Furthermore, the cell morphology study based on the cell semantic segmentation can be an interesting research direction.

## Supporting information

S1 Fig ROC curves for cell segmentation using M-UNet. ROC curves on the first testing region for the two specimens.
(TIF)
