# Supplementary figures and images for: Automatic cell counting from stimulated Raman imaging using deep learning
Source: PLoS One. 2021 Jul 21;16(7):e0254586. doi: 10.1371/journal.pone.0254586 (PMC8294532; doi:10.1371/journal.pone.0254586)

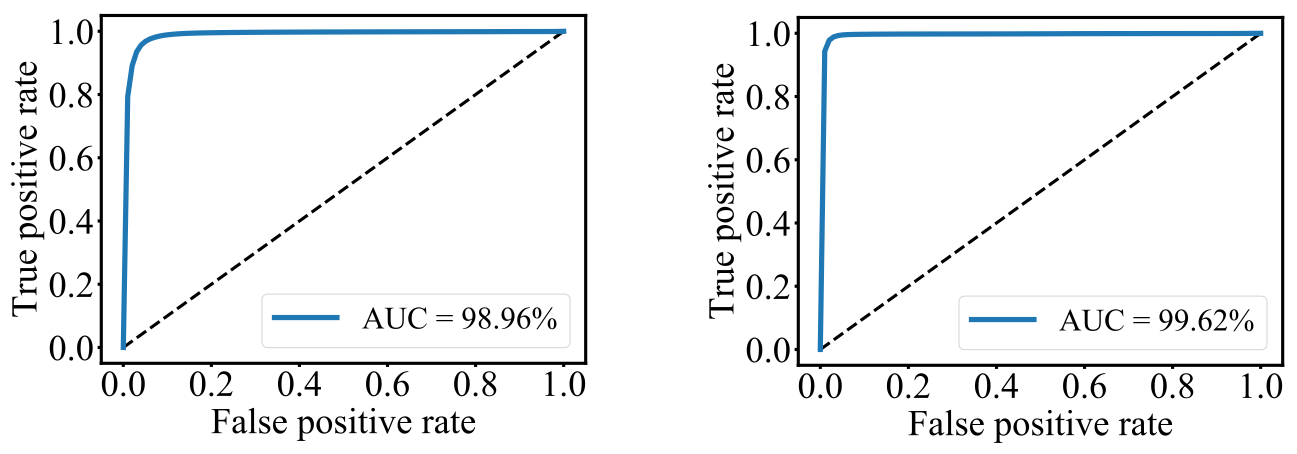

Supplement: S1 Fig — ROC curves on the first testing region for the two specimens. (TIF) [file pone.0254586.s001.tif]
