# Supplementary material for: Automatic cell counting from stimulated Raman imaging using deep learning
Source: PLoS One. 2021 Jul 21;16(7):e0254586. doi: 10.1371/journal.pone.0254586 (PMC8294532; doi:10.1371/journal.pone.0254586)
Supplement: Attachment — Submitted filename: Response to Reviewer.pdf [file pone.0254586.s002.pdf]

Responses to a Reviewer's Comments

Ref: Submission ID a14f1437-e3b9-416b-bed5-ddd84230a26e
Title: Automatic Cell Counting from Stimulated Raman Imaging Using Deep Learning
Journal: Plos One

We appreciate the effort and the constructive comments from the editors and reviewers. In response, we revised the manuscript accordingly. Text changes/additions to the manuscript are highlighted in red.

Reviewer #1 Comments

The manuscript subject is very interesting however needs some minor and major changes:

1. The text of the manuscript should be justified to give your text a clean, crisp edge to be more polished.

Thank you for the comment. The manuscript has been proofread by a professional proofreader. The confusing text and wordings have been corrected.

2. The introduction should not include any figures. Figure 1 should be added to the method section with related texts.

Thank you for the comment. Fig 1 has been moved to the Methodology Section.

Section 3. Methodology (lines 205 - 207, pg. 6):
Fig 1 shows SRS images from brain tumor samples. The cell bodies are shown with blue signals embedded in the fatty tissue background of green signals.

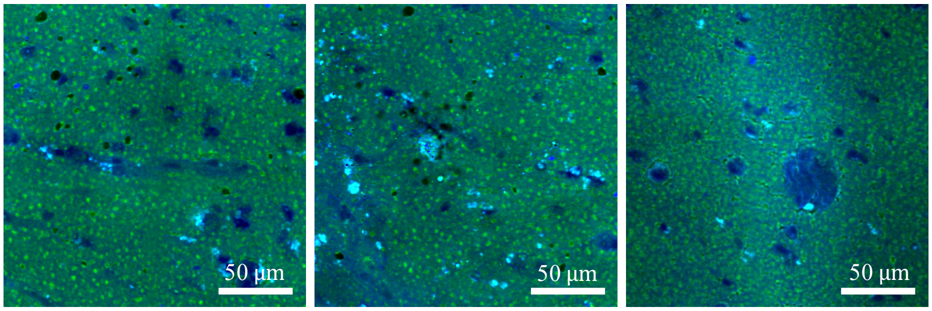

Fig 1. SRS Samples. Representative SRS images show cell bodies embedded in the fatty tissue background. Pseudocolor green: lipids; Pseudocolor blue: proteins.

3. It is important to add the powerful table of literature review for a comparison with other works and disadvantages of the literature.

Thank you for the comment. We have summarized the cell counting research in Table 1 and illustrated the difference between this research and the literature.

Table 1. A summary of the cell counting research.

Category	Authors	Method
Detection-based	Arteta et al. (2012)	SVM, dynamic programming, MSER detector
	Arteta et al. (2016)	Tree-structured discrete graphical model, SVM, dynamic programming
	Hosseini et al. (2020)	Mask R-CNN
Regression-based	Xue et al. (2016)	AlexNet, ResNet
	Xie et al. (2018)	Fully convolutional regression network
	Rad et al. (2018)	Ensemble residual dilated U-Net
	Villa et al. (2018)	CNN, LSTM
	Xue et al. (2019)	CNN, compressed sensing, sparse coding
	Xu et al. (2019)	U-Net
	He et al. (2019, 2021)	FCN, shortcut connections

Regarding the SRS image analysis, there are some attempts in the literature that integrated the ML technique, specifically, deep learning, into the analysis. Most research performed the lesion prediction task for image patches within a specimen [1, 2]. This research extends the simple image classification to pixel-level analysis by providing the cell segmentation and cell counting results that can reveal the intrinsic sample characteristics of brain tissues. Motivated by the superiority of U-Net over FCN on the medical image semantic segmentation task, this research employs and modifies U-Net to segment cells on SRS images. Also, the proposed cell counting scheme enables a mix of detection and regression-based counting because cells are segmented without the requirement of identifying each cell instance, but identified and counted through the involvement of morphological analysis.

4. In table 2 performance criteria of the classification method are presented however, what are the means of standard deviation or mean. Does each criterion have single parentage? Moreover, you should report other factors such as precision and negative predictive value, and fall-out.

Thank you for pointing this out. To show the cell segmentation results, the experiments are performed with five replications. Therefore, the means and standard deviation of means are the metrics calculated from the five replications. The description of the mean and standard deviation are highlighted in Subsection 4.1 Pixel-Wise Evaluation on the First Testing Region.

Section 4.1. Pixel-Wise Evaluation on the First Testing Region (lines 369 - 370, pg. 11):

Table 3 and 4 summarize the mean and standard deviation (SD) of the pixel-wise segmentation evaluation for the two specimens.

The calculation of sensitivity, precision, and are shown as follows:

$$\text{Sensitivity} = \frac{\text{TP}}{\text{TP} + \text{FN}} \tag{1}$$

$$\text{Precision} = \frac{\text{TP}}{\text{TP} + \text{FP}} \tag{2}$$

$$\text{F1 (DICE)} = \frac{2\text{TP}}{2\text{TP} + \text{FP} + \text{FN}} \tag{3}$$

where TP, TN, FP, and FN are the number of true positive, true negative, false positive and false negative, respectively. It can be referred that

$$\text{F1} = \frac{2\text{Precision} * \text{Sensitivity}}{\text{Precision} + \text{Sensitivity}} \tag{4}$$

$$\text{Precision} = \frac{\text{F1} * \text{Sensitivity}}{2\text{Sensitivity} - \text{F1}} \tag{5}$$

Also, fall-out is also known as false positive rate (FPR), which can be calculated by

$$\mathrm{FPR} = \frac{\mathrm{FP}}{\mathrm{TN} + \mathrm{FP}} \tag{6}$$

(7)

According to the definition of specificity

$$\mathrm{Specificity} = \frac{\mathrm{TN}}{\mathrm{TN} + \mathrm{FP}} \tag{8}$$

FPR can be calculated by

$$\mathrm{FPR} = 1 - \mathrm{Specificity} \tag{9}$$

(10)

Based on Equation (5) and Equation (9), precision and FPR can be referred from the performance metrics in Table 3 and Table 4. Because of the page limitation, the metrics are not provided in the manuscript.

5. Figure quality is very low and should be designed again in vector-based formats with software like adobe illustrator in PDF, SVG, EPS, or other vector formats. This type of figure is not acceptable for papers.

Thank you for the comment. The figures are generated with a high resolution and saved as pdf. The figures are damaged in the submission process. Please check the original file from the link on the figure page.

6. You should add the setup image and description of your experiment.

Thank you for the comment. We have highlighted the SRS image setup and the experimental settings in Section 4 Experimental Results. A more detailed description of SRS sample preparation can be found in Lu et al. (2016) [3].

Section 4. Experimental Results (lines 329 - 336, pg. 10):
In the experiment, brain tumor image samples are obtained from Lu et al. (2016) [3]. In particular, the brain tissues were collected from the Brigham and Women's Hospital and Dana-Farber Cancer Institute. A flash-freezing process was conducted at -80°C, followed by a sectioning process to 12-μm thicknesses. The brain tumor samples are imaged by SRS and then stained using the H&E technique. A non-neoplastic benign brain tumor specimen with epilepsy and a malignant anaplastic oligodendroglioma specimen are utilized to conduct the cell counting task. Specifically, the resolution for the two specimens are 0.37 μm/pixel and 0.18 μm/pixel, respectively.

Section 4. Experimental Results (lines 337 - 344, pg. 10):
The SRS image is split into three regions: one training region and two testing regions. It is noted that there are mismatches between the obtained H&E and SRS images for the same specimen regarding the cell shape, size, and position. Cell shiftiness and vanishing are also observed during the image collection process, which leads to a lack of ground truth cell distribution information. Therefore, the cells within the training region and the first testing region are annotated manually, which can be used to train the U-Net model and evaluate the cell segmentation results.

7. How you extract ground truth images. It should explain clearly and describe the accuracy of clustering techniques.

Thank you for the comment. The brain tumor samples are firstly imaged by SRS and then stained using the H&E technique. Therefore, we assume the number of cells

from the H&E images is the ground truth. Regarding cell segmentation, it is noted that there are mismatches between the obtained H&E and SRS images for the same specimen regarding the cell shape, size, and position. Cell shiftiness and vanishing are also observed during the image collection process, which leads to a lack of ground truth cell distribution information. Therefore, the cells within the training region and the first testing region for each specimen are annotated manually.

The clustering is conducted on H&E images. Because of the clear differentiation between cells and the background, which is generated in the staining process, the clustering technique can easily segment cells. A demonstration of H&E image and the corresponding clustering results are shown in Fig 6 (c) - (d). The assumption is that the cell segmentation results from H&E images are the ground truth with 100% accuracy.

Section 4. Experimental Results (lines 338 - 344, pg. 10):
It is noted that there are mismatches between the obtained H&E and SRS images for the same specimen regarding the cell shape, size, and position. Cell shiftiness and vanishing are also observed during the image collection process, which leads to a lack of ground truth cell distribution information. Therefore, the cells within the training region and the first testing region for each specimen are annotated manually, which can be used to train the U-Net model and evaluate the cell segmentation results.

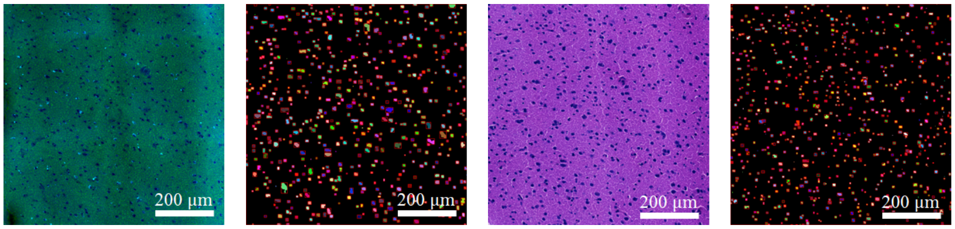

(a) **(b)** **(c)** **(d)**

Fig 6. Cell segmentation and identification results in a FOV. A: SRS FOV. B: SRS Cell identification. C: H&E FOV; D: H&E Cell identification.

8. Based on figure 4, the results of clustering are so weak, and large areas of the images are missed.

Thank you for the comment. Fig 4 is the post-morphological analysis for the connected cell regions, not the clustering result. The clustering results are shown in Fig 6 (d). The paragraph has been revised the address the case of connected cells.

Section 3.3. Cell Counting via Morphological Analysis (lines 296 - 301, pg. 9):
However, overlapping cells exist. In this case, multiple cells can be recognized as one region. Therefore, a post-morphological analysis that uses distance transform and watershed segmentation algorithms is further employed for each identified region, where connected cells can be split, which enhances the cell counting results [4]. Fig 4 presents two samples of connected cells, which are further split using the distance-based watershed segmentation method.

9. The data set is very small for the claim.

Thank you for pointing this out. The dataset in this research is adopted from Lu et al. (2016) [3] by selecting the samples with good conditions for cell counting. Lu et al. performed brain tumor imaging using SRS and established the methodology of real-time brain tissue image collection in clinical. Based on the prior conclusions from Lu et al. (2016), this research further focused on the application of SRS in practical and claimed

that AI can perform SRS image analysis in a detailed level and enhance the potential of promoting the SRS technique into the surgical process. Currently, due to the limited application of the SRS technique, the dataset is the only data available for brain tumor. Therefore, we only apply the cell counting to the dataset, which includes 5388 image patches in total. The related statements are shown as follows:

Section 1. Introduction (lines 87 - 91, pg. 3):
This research not only demonstrates the possibility of performing SRS image analysis in a much more detailed level but also enhances the potential of promoting the SRS technique into the surgical process, which quickly provides surgical guidance without the requirement of the time-consuming stain process.

Section 4. Experimental Results (lines 423 - 424, pg. 13):
This study aims to promote the implementation of AI to biomedical analysis for SRS images.

10. You should also report the ROC curve for segmentation.

Thank you for the comment. We didn't include the ROC curves in the manuscript because of the page limitation. We have added the ROC curves in the Appendix.

Section S1 Appendix. (lines 445 - 446, pg. 14):

S1 Appendix. ROC curves for cell segmentation using M-UNet.

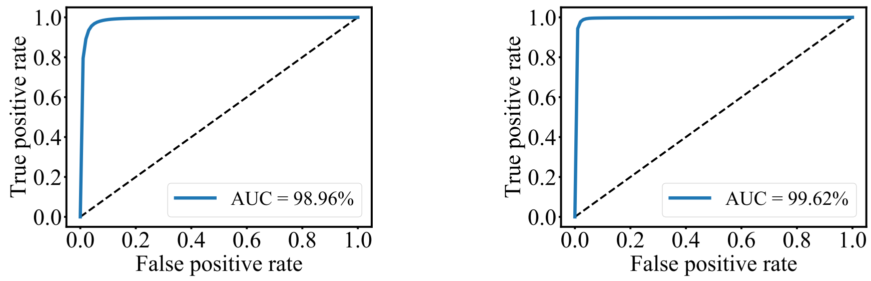

(a) Specimen 1 **(b)** Specimen 2

Fig 9. ROC curves on the first testing region for the two specimens.

11. The results are so weak it should be developed and used powerful papers for description.

Thank you for the suggestion. Other than FCN and U-Net, image segmentation methods include regional convolutional network (R-CNN), dilated convolutional models, recurrent neural network, attention-based models, generative models, etc [5]. The models have different advantages and applications. For instance, R-CNN was proposed for object detection for complicated images. Attention enables an understanding of the relationship between different positions and scales within an image. Although they achieved promising performance, those models require a great number of training samples and complicated model architectures. Regarding cell segmentation for medical images, due to the simple cell structure and the lack of available annotation information, most research applied simple model structures such as FCN and U-Net [6–10]. Therefore, we modified the U-Net model and compared it to the conventional U-Net and FCN. Also, this research focuses more on the application of the deep learning technique to solve practical problems in the surgical process efficiently from an application perspective, instead of showing the modified U-Net as the best model theoretically. As

stated in the Introduction section, this study aims to demonstrate the possibility of performing SRS image analysis using deep learning and enhance the potential of promoting the SRS technique into the surgical process from an application perspective.

Section 1. Introduction (lines 87 - 91, pg. 3):
This research not only demonstrates the possibility of performing SRS image analysis in a much more detailed level but also enhances the potential of promoting the SRS technique into the surgical process, which quickly provides surgical guidance without the requirement of the time-consuming stain process.

12. Therefore, it seems that your manuscript needs additional evaluation and comparison with other works moreover, the implemented code should be presented in the GitHub.

Thank you for the comment. We have uploaded the implementation code to https://github.com/vestal-doublekuan/SRS-image-cell-counting .

Reviewer #2 Comments

1. It would be nice for the authors to provide the reasons to include two images in Fig 1 under the same magnification, 50 µm.

Thank you for pointing this out. We have modified the images in Fig 1 with the same magnification.

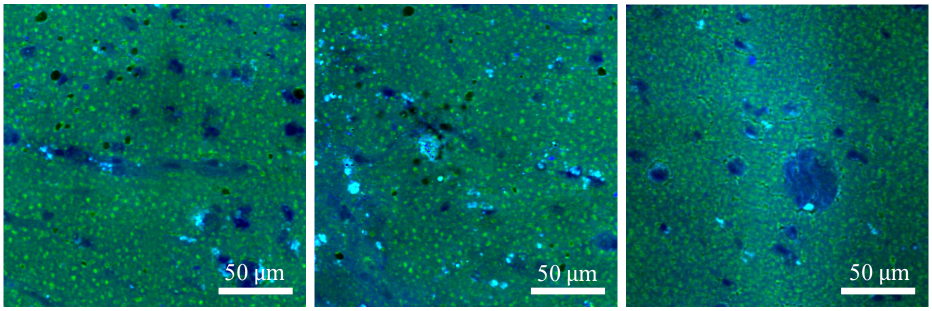

Fig 1. SRS Samples. Representative SRS images show cell bodies embedded in the fatty tissue background. Pseudocolor green: lipids; Pseudocolor blue: proteins.

2. The text within the U-Net in Fig 2 are not readable due to low resolution.

Thank you for the comment. We have enlarged the texts within the figure.

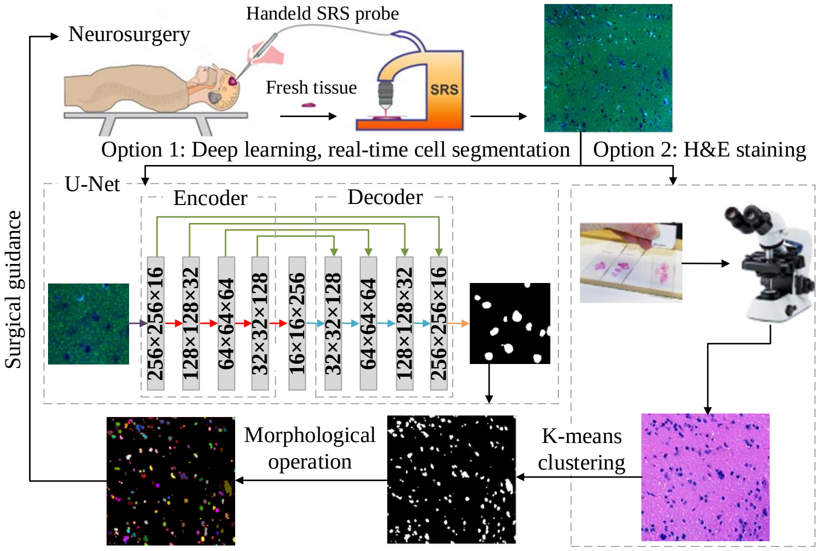

Fig 2. Overview of the cell counting framework.

3. The modified U-Net essentially used a set of different hyper-parameters to alter the network structure from the original. Is there any other novel contribution applied to the network?

Thank you for the comment. This research focuses on the application of U-Net on a new medical image modality for cell counting. The differences between M-UNet and the original U-Net include the number of kernels involved, the number of input color channels, and the application of the early-stopping technique. The modification is

motivated by the simple SRS image color range that does not require the original U-Net structure due to the model complexity. The simplified M-UNet can also improve the model training efficiency.

4. The authors might want to compare the performance of U-Net with some other models' performances. Please see the article "Image Segmentation using Deep Learning: A survey" for detail.

Thank you for the suggestion. Minaee et al. (2021) summarized image segmentation methods comprehensively. Other than FCN and U-Net, other methods include regional convolutional network (R-CNN), dilated convolutional models, recurrent neural network, attention-based models, generative models, etc. The models have different advantages and applications. For instance, R-CNN was proposed for object detection for complicated images. Attention enables an understanding of the relationship between different positions and scales within an image. Although they achieved promising performance, those models require a great number of training samples and complicated model architectures. Regarding cell segmentation for medical images, due to the simple cell structure and the lack of available annotation information, most research applied simple model structures such as FCN and U-Net [6–10]. Therefore, we modified the U-Net model and compared it to the conventional U-Net and FCN. Also, as stated in the Introduction section, this study aims to demonstrate the possibility of performing SRS image analysis using deep learning and enhance the potential of promoting the SRS technique into the surgical process from an application perspective.

Section 1. Introduction (lines 87 - 91, pg. 3):

This research not only demonstrates the possibility of performing SRS image analysis in a much more detailed level but also enhances the potential of promoting the SRS technique into the surgical process, which quickly provides surgical guidance without the requirement of the time-consuming stain process.

Reviewer #3 Comments

1. Authors need to explain the difference between the 5-layers U-Net and M-Net.

Thank you for the comment. The 7layer-UNet and 5layer-UNet are implemented and compared to the M-UNet to confirm the necessity of the U-Net structure. As shown in Fig 3, the M-UNet and original U-Net can be referred as 9layer-UNet. The main difference between 7layer-UNet, 5layer-UNet, and M-UNet is the number of convolutional layers. In particular, 7layer-UNet removes one encoder block and one decoder block, whereas 5layer-UNet removes two encoder blocks and two decoder blocks. The difference between the three structures is addressed in Section 4.1 Pixel-Wise Evaluation on the First Testing Region (lines 382 - 385, pg. 11).

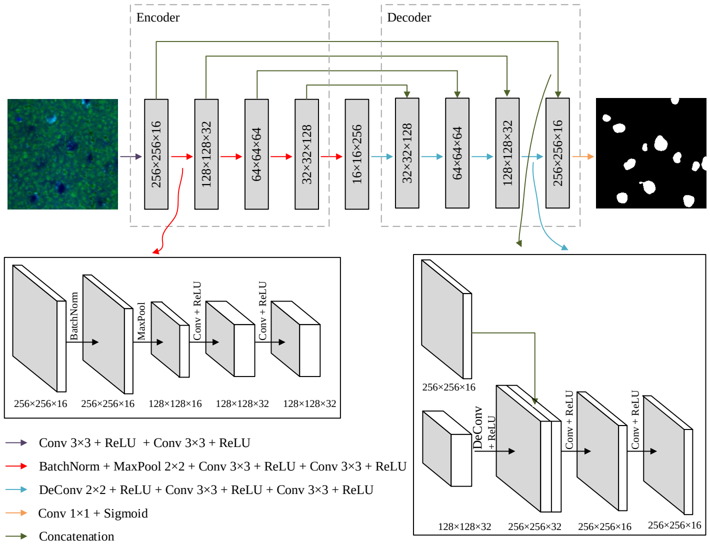

Fig 3. The architecture of the modified U-Net model.

Section 4.1 Pixel-Wise Evaluation on the First Testing Region (lines 382 - 385, pg. 11):

To confirm the necessity of the U-Net structure, the simplified U-Net that removes one encoder block and one decoder block is performed as 7layer-UNet. Two blocks of both encoders and decoders are also excluded as 5layer-UNet in the experiment.

2. In line 317-327, authors need to specify the maximum size of the cell.

Thank you for the comment. We have added maximum cell size in the manuscript.

Section 4.5 (lines 327, pg. 9):

In this case, the maximum cell obtained is 375 μm^2.

3. Authors need to justify why the training time difference is too much between 5-layer U-Net and M-Net.

Thank you for pointing this out. The time difference is caused by different machines in the experiments by mistake. To correct this, we have replaced the training time with the number of epochs employed. In the training process, if the validation loss has not been reduced for 25 epochs, the training process will stop. Also, U-Net was trained

without early stopping, the default number of epochs is 300.

Section 3.1 Cell Segmentation Using U-Net Based on SRS images (lines 272 - 273, pg. 8):

If the validation loss has not been reduced for 25 epochs, the training process will stop.

Table 3. Cell segmentation results on the first testing region for Specimen 1.

Performance	M-UNet		U-Net		7layer-UNet		5layer-UNet		FCN	
Metrics	Mean	SD	Mean	SD	Mean	SD	Mean	SD	Mean	SD
Accuracy(%)	97.69	0.23	97.62	0.11	97.82	0.10	97.84	0.05	97.83	0.11
Sensitivity(%)	85.57	1.79	81.43	1.73	82.41	3.24	82.50	1.95	83.40	2.14
Specificity(%)	98.48	0.33	98.69	0.13	98.84	0.25	98.86	0.13	98.79	0.23
AUC(%)	98.96	0.16	98.59	0.22	98.94	0.11	98.92	0.10	99.02	0.14
DICE(%)	81.99	1.36	80.96	0.92	82.43	0.81	82.61	0.55	82.74	0.54
Epochs	60.40	5.18	300.00	0.00	60.20	4.76	77.80	16.12	65.20	6.61
PE(%)	2.49	1.36	2.51	2.08	3.45	4.73	3.26	1.62	4.07	2.64

Table 4. Cell segmentation results on the first testing region for Specimen 2.

Performance	M-UNet		U-Net		7layer-UNet		5layer-UNet		FCN	
Metrics	Mean	SD	Mean	SD	Mean	SD	Mean	SD	Mean	SD
Accuracy(%)	99.11	0.10	99.15	0.05	98.88	0.05	98.55	0.04	99.12	0.05
Sensitivity(%)	87.64	1.34	87.78	1.22	86.12	2.39	74.14	2.65	86.47	2.83
Specificity(%)	99.54	0.13	99.57	0.05	99.36	0.14	99.45	0.11	99.58	0.09
AUC(%)	99.62	0.09	99.66	0.09	99.58	0.07	99.21	0.08	99.55	0.13
DICE(%)	87.56	1.21	88.11	0.75	84.66	0.33	78.47	0.65	87.47	0.84
Epochs	78.40	13.24	300.00	0.00	95.00	24.05	144.40	12.76	115.40	14.36
PE(%)	3.35	2.41	4.16	2.52	8.90	2.68	4.86	4.15	4.39	3.63

4. The proposed M-Net lacks architectural difference compared to 5 layers U-Net.

Thank you for the comment. The 7layer-UNet and 5layer-UNet are implemented and compared to the M-UNet to confirm the necessity of the U-Net structure. As shown in Fig 3, the M-UNet and original U-Net can be referred as 9layer-UNet. The main difference between 7layer-UNet, 5layer-UNet, and M-UNet is the number of convolutional layers. In particular, 7layer-UNet removes one encoder block and one decoder block, whereas 5layer-UNet removes two encoder blocks and two decoder blocks. The difference between the three structures is addressed in Section 4.1 Pixel-Wise Evaluation on the First Testing Region (lines 382 - 385, pg. 11).

Section 4.1 Pixel-Wise Evaluation on the First Testing Region (lines 372 - 375, pg. 11):

To confirm the necessity of the U-Net structure, the simplified U-Net that removes one encoder block and one decoder block is performed as 7layer-UNet. Two blocks of both encoders and decoders are also excluded as 5layer-UNet in the experiment.

References

1. Orringer DA, Pandian B, Niknafs YS, Hollon TC, Boyle J, Lewis S, et al. Rapid intraoperative histology of unprocessed surgical specimens via fibre-laser-based

stimulated Raman scattering microscopy. Nature Biomedical Engineering. 2017;1(2):0027.

2. Zhang L, Wu Y, Zheng B, Su L, Chen Y, Ma S, et al. Rapid histology of laryngeal squamous cell carcinoma with deep-learning based stimulated Raman scattering microscopy. Theranostics. 2019;9(9):2541–2554.

3. Lu FK, Calligaris D, Olubiyi OI, Norton I, Yang W, Santagata S, et al. Label-free neurosurgical pathology with stimulated Raman imaging. Cancer Research. 2016;76(12):3451–3462.

4. Vincent L, Soille P. Watersheds in digital spaces: an efficient algorithm based on immersion simulations. IEEE Transactions on Pattern Analysis & Machine Intelligence. 1991;(6):583–598.

5. Minaee S, Boykov YY, Porikli F, Plaza AJ, Kehtarnavaz N, Terzopoulos D. Image segmentation using deep learning: A survey. IEEE Transactions on Pattern Analysis and Machine Intelligence. 2021;.

6. Xie W, Noble JA, Zisserman A. Microscopy cell counting and detection with fully convolutional regression networks. Computer Methods in Biomechanics and Biomedical Engineering: Imaging & Visualization. 2018;6(3):283–292.

7. Rad RM, Saeedi P, Au J, Havelock J. Blastomere cell counting and centroid localization in microscopic images of human embryo. In: 2018 IEEE 20th International Workshop on Multimedia Signal Processing (MMSP). IEEE; 2018. p. 1–6.

8. Xu Z, Sobhani F, Moro CF, Zhang Q. US-net for robust and efficient nuclei instance segmentation. arXiv preprint arXiv:190200125. 2019;.

9. He S, Minn KT, Solnica-Krezel L, Anastasio M, Li H. Automatic microscopic cell counting by use of deeply-supervised density regression model. In: Medical Imaging 2019: Digital Pathology. vol. 10956. International Society for Optics and Photonics; 2019. p. 109560L.

10. He S, Minn KT, Solnica-Krezel L, Anastasio MA, Li H. Deeply-supervised density regression for automatic cell counting in microscopy images. Medical Image Analysis. 2021;68:101892.
